# Elsberg Syndrome with Mixed Presentation as Meningitis Retention Syndrome: A Pediatric Case Report and Comprehensive Review of the Literature

**DOI:** 10.3390/children10040724

**Published:** 2023-04-14

**Authors:** Mandy Hsu, Nader El Seblani, Zahra Zhu, Bhuvaneswari Ramisetty, Christopher Day, Jikku Zachariah, Divpreet Kaur, Ashutosh Kumar, Sita Paudel, Dustin Paul, Puneet Singh Kochar, Paul R Carney, Sunil Naik

**Affiliations:** 1 University Park Program, Penn State College of Medicine, State College, PA 16801, USA; 2 Department of Neurology, Milton S. Hershey Medical Center, Penn State College of Medicine, Hershey, PA 17033, USA; 3 College of Medicine, Penn State University, Hershey, PA 17033, USA; 4 Alluri Sita Ramaraju Academy of Medical Sciences, Eluru 534005, India; 5 Department of Pediatrics and Neurology, Penn State Children’s Hospital, Penn State College of Medicine, Hershey, PA 17033, USA; 6 Department of Radiology, Division of Neuroradiology, Penn State College of Medicine, Hershey, PA 17033, USA; 7 Division of Pediatric Neurology, Department of Child Health, The University of Missouri at Columbia, Columbia, MO 65211, USA

**Keywords:** Elsberg syndrome, meningitis retention syndrome (MRS), encephalopathy, West Nile virus, urinary neuroanatomy, urinary retention, meningoencephalitis, myeloencephalitis

## Abstract

Elsberg syndrome is a typically infectious syndrome that may cause acute or subacute bilateral lumbosacral radiculitis and sometimes lower spinal cord myelitis. Patients often present with various neurological symptoms involving the lower extremities, including numbness, weakness, and urinary disturbances such as retention. A 9-year-old girl with no significant past medical history presented with altered mental status, fever, urinary retention, and anuria and was found to have encephalomyelitis. An extensive diagnostic workup led to ruling out possible etiologies until identifying Elsberg syndrome. In this report, we describe a case of Elsberg syndrome caused by West Nile virus (WNV). To the best of our knowledge, this is the first reported case of its kind in the pediatric population. Utilizing PubMed and Web of Science databases, we reviewed the literature to describe the neurogenic control of the urinary system in correlation to a multitude of neurologic pathologies.

## 1. Introduction

### Elsberg Syndrome

Elsberg syndrome (ES) is a syndrome of acute or subacute bilateral lumbosacral radiculitis presumed to be caused by infection, which is often a reactivation or manifestation of herpes simplex virus 2 (HSV2) [1]. Most literature detailing Elsberg syndrome to date consist of case studies describing the syndrome in association with infectious diseases, such as HSV2, varicella-zoster virus, human immunodeficiency virus, and COVID-19 [2,3,4,5], as well as treatments, such as the immunosuppressives used to treat multiple sclerosis (MS) predominantly in adults. Recent articles have suggested that Elsberg syndrome is a less-recognized cause of cauda equina syndrome [4,6], a syndrome where lumbar and sacral nerve roots in the cauda equina are dysfunctional. However, there is very limited information about Elsberg syndrome in pediatric populations. To date and to the best of our best knowledge, only one 2007 article describes a case of a 21-month-old boy presenting with urinary retention that was secondary to aseptic meningitis, who demonstrated vesicorectal disturbance and neurologic symptoms similar to Elsberg syndrome in adults [7].

Elsberg syndrome (ES) typically presents with a constellation of symptoms related to sacral radiculitis and lower spinal cord myelitis, which include urinary retention, lower extremity paresthesia, flaccid paresis, and constipation [1]. It is usually associated with a primary infection or reactivation of herpes simplex virus type 2 (HSV-2) [8]; however, there have been case reports of varicella zoster [9] and SARS-COV-2 [2] causing ES. Though ES is an under-considered cause of cauda equina syndrome (CES), in a recent study, ES was found to be the cause of CES in 5–15% of cases [1].

Patients presenting with headache, acute encephalopathy, progressive greater bilateral upper extremities, and urinary retention can have a broad differential diagnosis such as autoimmune encephalomyelitis, Guillain–Barré syndrome (GBS), Bickerstaff encephalitis, and acute disseminated encephalomyelitis (ADEM). Other differentials include sarcoidosis, lymphoma, other neoplasms, Lyme disease, and dural arteriovenous fistula (AVF).

## 2. Case Report

A 9-year-old girl with no significant past medical history presented with altered mental status, urinary retention, and anuria. Two weeks prior to her presentation, the patient and her family had visited friends in Michigan, USA, for a week, where the patient enjoyed some swimming activities in a local pond and suffered from mosquito bites on her back. A couple of days later, the patient started having pain in her lower extremities followed by neck pain. There were no symptoms of sore throat, cough, or runny nose. The family later moved to a local camping site in Pennsylvania, USA, about a week later, where the patient started to become lethargic, complaining of headache with photophobia, nausea, vomiting, and abdominal pain with one spike of low-grade fever. The patient was evaluated in an urgent care clinic and found to be drowsy, tachycardic, tachypneic, and febrile with low urine output, for which she was sent to the emergency department (ED) for further evaluation.

On her ED presentation, the patient was febrile, nauseous, tachycardic, and tachypneic but with normal pulmonary breath sounds. She had a scattered pinpoint red/purple non-blanchable rash on her bilateral feet, dorsum, and palmar surfaces of her hands. On limited neurological examination, she was alert and oriented to self, place, and time but was very lethargic and somnolent. She did not communicate verbally, and had her eyes closed most of the time. She had intact bilateral visual fields and her pupils were reactive to light, but she demonstrated bilateral mydriasis with bilateral horizontal slow beating nystagmus. Her motor examination was remarkable, with significantly more weakness in the bilateral lower extremities than upper extremities. She had brisk reflexes in the upper and right lower extremities but diminished left patellar and Achilles reflexes. She had normal flexor plantar and Hoffman’s responses. Her sensations were intact to light touch throughout her body but without withdrawal to noxious stimuli to her feet. Because the patient had severe urinary retention, a Foley catheter was placed, resulting in 1300 mL of urine output.

Her initial lab workup was remarkable for neutrophilic predominant leukocytosis with a white blood cell (WBC) count of 18,000/µL, elevated anion gap with metabolic acidosis of HCO3 13 mmol/L, blood glucose 186 mg/dL, normal liver function tests, and A1C 5.4%. She had a normal erythrocyte sedimentation rate, C-reactive protein, and procalcitonin, and a negative respiratory virus panel. She also had a normal chest X-ray and CT brain without contrast. Her encephalopathy, fever, lower extremities weakness, and urinary retention raised concerns for myeloencephalitis, for which the patient was admitted to the pediatric intensive care unit and started on Ceftriaxone, Vancomycin, and Doxycycline. Initial brain MRI/MRA and whole spine MRI with and without contrast demonstrated no acute intracranial abnormalities on the initial reading; however there was a subtle contrast enhancement of the distal thoracic cord and cauda equina. Cerebrospinal fluid (CSF) studies revealed elevated opening pressure at 35 mm H_2_O, 2 nucleated cells, 1 red blood cell, glucose 71, protein 31, elevated myelin basic protein, and no elevated oligoclonal bands. Given that she demonstrated elevated intracranial pressure (ICP) on her ICP monitoring devices, along with worsening tachypnea, encephalopathy, and ophthalmoplegia, the patient was intubated for airway protection and a lumbar drain was inserted. Lyme disease, enterovirus, herpes simplex virus PCR, legionella antigen, Epstein–Barr virus (EBV), and enteric pathogen panel were negative, but cytomegalovirus testing was indicative of prior infection. Enteric pathogen panel including Campylobacter, Parvovirus B-19 (IgM, IgG, and PCR), Bartonella Henselae (IgM and IgG), serum Eastern Equine encephalitis (EEE), Powassan virus, EBV, and Rickettsia serology tests were all negative. Autoimmune encephalitis panels, anti-Gq1b antibodies, NMO/MOG and MS CSF, and serum studies were also negative. Furthermore, autoimmune encephalopathy panels (both CSF and serum) were negative.

The patient’s encephalopathy and weakness continued to worsen. Given that autoimmune encephalomyelitis, Guillain–Barré syndrome (GBS), and Bickerstaff encephalitis were on the differential, the patient was empirically treated with IVIG.

Repeat CSF studies a few days later showed mild pleocytosis of 11/µL nucleated cells, 820/µL red blood cells, 57 mg/dL glucose (normal: 60–80 mg/dL), and 27 mg/dL proteins (normal: 15–45 mg/dL). Antibiotics were discontinued except for doxycycline, which was continued for a total of 7 days.

**Figure 1:**
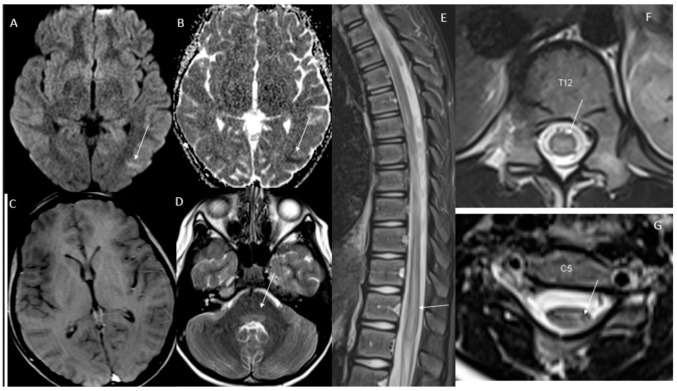
Initial axial DWI/ADC maps demonstrate subtle small area of restricted diffusion in left posterior temporal lobe (white arrows in images **A**,**B**) and without perceivable enhancement (image **C**). There is additional subtle T2 prolongation in the dorsal pons in the midline and paramedian region (white arrow image **D**). Initial sagittal STIR image of the thoracic spine demonstrates scattered patchy areas of T2 prolongation with worst involvement of the lower cord and conus medullaris (white arrows in images **E**,**F**). Additional patchy T2 prolongation is seen in left lateral cervical spinal cord at C5 (white arrow in image **G**).

**Figure 2:**
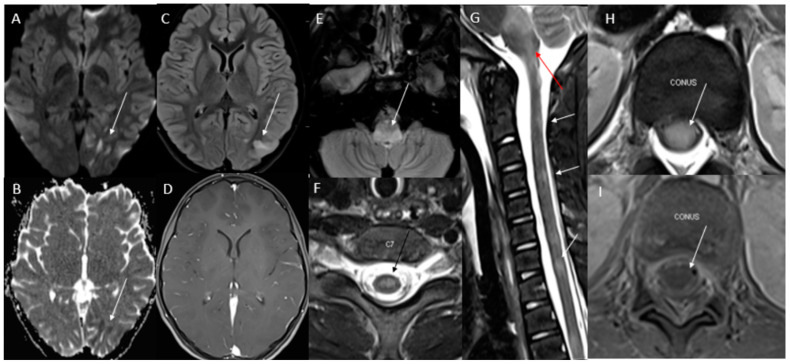
Repeat neuroaxis imaging shows increasing patchy restricted diffusion in left posterior temporal and occipital lobes (white arrows in images **A**,**B**). Small area in left posterior temporal lobe which demonstrated restricted diffusion on prior MRI brain shows increasing T2 prolongation (white arrow in image **C**). There is no perceivable enhancement (image **D**). Repeat imaging showing dorsal medulla in the expected location of area postrema (white arrow in image **E**). Axial T2 image through the lower cervical spinal cord demonstrates cord swelling and central cord T2 prolongation (black arrow in image **F**). Sagittal STIR image of the cervical spine shows multifocal areas of T2 prolongation with cord swelling (white arrow in image **G**) and is also noted in extensive T2 prolongation in the dorsal brain stem (red arrow image **G**). Axial T2 image through the conus medullaris demonstrates T2 prolongation with cord swelling (white arrow in image **H**) but without perceivable enhancement (white arrow in image **I**).

Initial axial diffusion weighted imaging (DWI)/apparent diffusion coefficient (ADC) showed a small area in the left posterior temporal lobe with restricted diffusion without T2 prolongation. There was T2 prolongation in the dorsal pons in the midline and paramedian region, thoracic spine, and left and lateral cervical spinal cord at C5 (Figure 1). Repeat brain and spine MRI/MRA after 3 days showed new T2 hyperintensities, hyperintensities in L/T/C spine with new areas of flair hyperintensities in the brainstem (medulla and area postrema), and left parietal–temporal hyperintensities with restricted diffusion but no enhancement (Figure 2). A trial of 1 g of IV methylprednisolone was given for 3 days with no major improvement.

Neurophysiological testing such as EMG/NCS and routine EEG studies were also unremarkable. The lumbar drain was later discontinued after 8 days and the patient received plasma exchange. The arbovirus antibody panel was positive for WNV IgG, 1.58 (negative < 1.29), and 0.03 for IgM (positive > 0.89), which could be indicative of a previous or current infection.

The patient was then started on PLEX and eventually started to express increased mobility, dexterity, and cognition. Her eye movements became more conjugated and participated more with the physical therapist. Shortly afterwards, she was extubated. Repeat WNV IgG serology in two weeks showed markedly reduced levels (0.25). Her urinary control also started to improve gradually, as she was able to spontaneously void and pass bowel movements. Overall, she continued to improve clinically, and she was eventually discharged to acute rehabilitation after 4.5 weeks of hospital stay.

The patient was noted to have subtle lower extremity weakness with normal reflexes during follow-ups. During her last clinic visit, she had normal strength in the lower extremities and was able to walk and run well.

She underwent a urodynamics study, which showed terminal detrusor overactivity and inefficient Valsalva voiding. She was started on Ditropan and clean intermittent catheterization. The patient’s parents felt that Ditropan helped significantly with the patient’s urinary urgency and accidents. Her bladder was managed with clean intermittent catheterization carried out twice daily after voiding. She was dry between voids and catheterizations. For her bowels, she took laxatives as needed and had no issues with fecal incontinence.

To the best of our knowledge, this case is being presented as the first pediatric case of ES with myeloencephalitis caused by West Nile virus with overlapping features of Elsberg syndrome (ES) and meningitis retention syndrome (MRS). Moreover, unique to this case is the rapid clinical improvement of the patient after plasmapheresis. The diagnosis of WNV was confirmed with higher IgG titers. MRI of the spine showed cord thickening and enhancement in the distal cord and cauda equina consistent with ES. Brain MRI showed temporal and brain stem enhancements consistent with meningoencephalitis.

## 3. Discussion

### 3.1. Methods

A narrative literature review was conducted by searching for keywords including “Elsberg syndrome”, “meningitis retention syndrome”, “MRS”, “radiculitis with urinary retention”, “neurological control of bladder”, “higher centers of bladder control”, “spinal cord lesions affecting micturition”, “pathological lesions of brain affecting micturition”, “stroke and bladder control”, “transverse myelitis”, “epidemiology of West Nile virus”, and “clinical features of West Nile virus” in PubMed and Web of Science. Articles relevant to these topics were manually selected and relevant information was extracted for analysis.

### 3.2. Neuroanatomy

**Figure 3:**
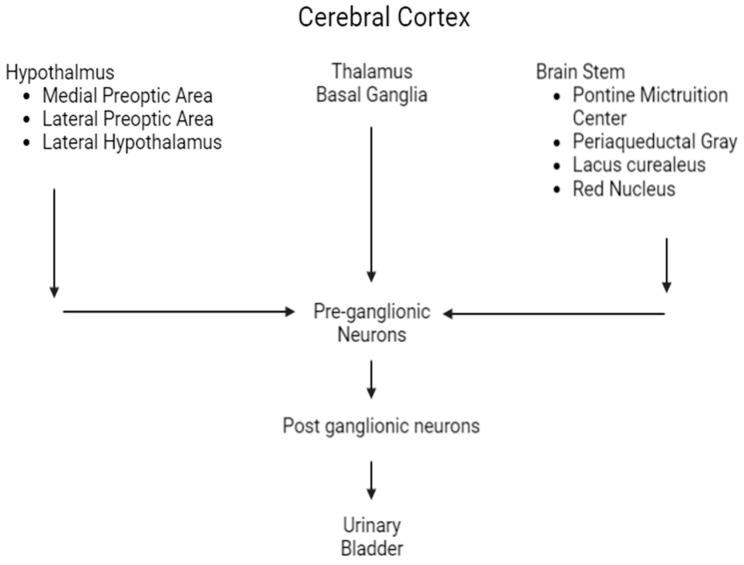
Brain structures involved with micturition.

**Figure 4:**
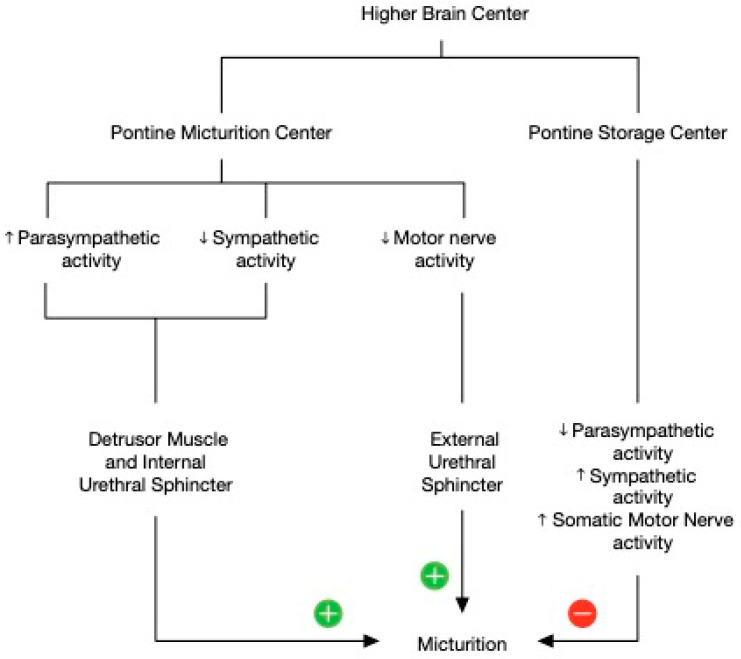
Activity and control involved during micturition.

The urinary bladder functions as a reservoir, storing urine and providing continence; this coordination is maintained by urinary bladder muscles and the urethral sphincter [10], which are controlled by higher brain centers. In the brain, micturition centers exist throughout the cerebral cortex [11], specifically in the prefrontal cortex, medial frontal cortex, insula, anterior cingulate gyrus, and stria terminalis. The centers of subcortical structures include the limbic system, medial preoptic area, lateral preoptic area, and paraventricular nucleus of the hypothalamus, thalamus, and basal ganglia. Brain stem areas involved are Barrington’s nucleus (PMC), the periaqueductal gray (PAG) of the midbrain, and the lateral pontine nuclei. These areas project to autonomic nuclei of the spinal cord and sphincter motor nuclei [12] regulating urinary continence. The serotonergic neurons of the medullary raphe nuclei, noradrenergic neurons of the locus coeruleus, and the noradrenergic A5 cell group of the brain stem have diffuse spinal projections necessary for the micturition reflex (Figure 3). The release of acetylcholine by somatic nerve terminals to skeletal muscle-type nicotinic muscle receptors also helps in maintaining urinary continence (Figure 4).

### 3.3. Peripheral Innervation of the Urinary Tract

Sensory impulses regarding bladder fullness are carried along the pelvic nerve, iliohypogastric nerves (T12-L1) [13], and the pudendal nerve (S2–S4). Sympathetic outflow arises from the thoracolumbar segments [14] (T12-L1) carrying noradrenergic signals. In contrast, parasympathetic outflow arises from sacral segments [15] (S2–S4) of the spinal cord carrying a cholinergic signal which acts on bladder muscle, bladder ganglion [16], and urethra preventing micturition. Somatic outflow is innervated by the pudendal nerve [17] (S2–S4). These pathways innervate the external urethral sphincter necessary for the voluntary control of continence [18].

A recent integrative model for the neurological control of the bladder states that the bladder is controlled by three separate brain “circuits” that connect to a central hub [19,20]. Circuit one is depicted as the typical circuitry for carrying bladder sensation and includes the thalamus, insula, and lateral prefrontal cortex, which leads to the medial prefrontal cortex. This is where voiding is controlled. The dorsal anterior cingulate cortex (dACC) and supplementary motor area (SMA) are involved in circuit two, which is crucial for producing a sense of urgency and managing the pelvic floor muscles to prevent unintentional urination in individuals with urge urine incontinence. The parahippocampal gyrus is assumed to be involved in circuit three, while its specific functional role is unknown.

## 4. Mechanism of Urinary Retention, Potential Pathologies, and Localization That May Occur along the Pathways

### 4.1. Ischemic Stroke and Bladder Control

Urodynamic studies of ischemic stroke patients who have subsequently developed urinary symptoms have shown that voiding is normally coordinated, that there is no evidence of detrusor sphincter dyssynergia, and that detrusor hyperreflexia is the pathologic finding [10].

Ischemic strokes that affect the anteriomedial frontal lobe, its descending pathway, and basal ganglia often cause the micturitional dysfunction that may be seen in stroke patients. Recently, Sakakibara and colleagues [21] found that in patients admitted for acute ischemic stroke, 3 months after the ischemic stroke, 53% of patients endured urinary complaints, 36% had nocturnal urinary frequency, 29% had urge incontinence, and 25% had difficulty voiding. Urinary retention was seen in the acute phase of illness in 6% of patients.

### 4.2. Basal Ganglia and Bladder Control

The basal ganglia is thought to have a net inhibitory effect on micturition. The micturition reflex is influenced by both dopamine (inhibitory in D1 and facilitatory in D2) and GABA (inhibitory). The dopamine D1-GABAergic direct pathway seems to be activated by the substangia nigra pars compacta neuronal firing and released striatal dopamine. This inhibits the basal ganglia output nuclei and, potentially, the micturition reflex via the GABAergic collateral to the micturition circuit. Along with the nigrostriatal fibers, the ventral tegmental area (VTA) may also be involved with the control of micturition.

In Parkinson’s disease, bladder symptoms may present after many years of treatment [11]. Patients may experience symptoms of urinary urgency, urinary frequency, as well as urge incontinence. Studies have also shown that Parkinson’s disease patients often experience detrusor hyperreflexia as the most common urodynamic abnormality [12].

### 4.3. Brainstem Lesion and Bladder Control

Sakakibara and colleagues [13] studied the urinary symptoms of 39 patients who had brainstem strokes. Almost half of the patients demonstrated urinary symptoms. Nocturnal urinary frequency was seen in 28% of patients, while urinary retention was seen in 21% of patients. A total of 8% of patients had urinary incontinence. It is likely that these problems were more common following hemorrhage because the damage was usually bilateral. Those with midbrain lesions did not demonstrate any urinary symptoms in this study. However, 35% and 18% of patients with pontine lesions and medullary strokes respectively did have urinary symptoms.

### 4.4. Spinal Cord Injury and Bladder Control

The sacral cord is connected to the pontine micturition centers via trans-spinal pathways. These connections are especially important for the detrusor and sphincter to be able to alternate between storage and voiding functions. When there is disconnection from the pons, the sphincter and detrusor muscle contract, causing a condition called “detrusor-sphincter dyssynergia”.

### 4.5. Spinal Cord Disease and Bladder Control

- Inflammatory
  - *Transverse myelitis (TM):* TM is a rare, acquired focal inflammatory disorder that often presents with symptoms such as bowel and bladder dysfunction, deficits in sensation, and rapid onset weakness. Inflammation may occur at any level of the spinal cord, but most commonly, it will affect the thoracic region of the spinal cord and lead to bilateral deficiencies. Less commonly, it can cause partial or asymmetric involvement. Idiopathic, postinfectious, systemic inflammation, and multifocal central nervous system disease are common etiologies of TM. The most common etiology of TM is idiopathic. Enteroviruses, West Nile virus, human immunodeficiency virus (HIV), human T-cell leukemia virus type 1 (HTV-1), herpes virus, neuroborreliosis (Lyme), Zika virus, *Mycoplasma,* and *Treponema pallidum* are just a few examples of infectious causes that may lead to TM [22].
    MS, neuromyelitis optica spectrum disorder (NMO), and ADEM are a few other conditions that may cause acquired central nervous system autoimmune disorders. The presentation for these diseases may be acute or subacute. Motor, sensory, and autonomic dysfunction are common neurologic symptoms of these diseases. Motor deficits, such as rapidly growing paraparesis, are sometimes caused by damage to spinal cord white matter. This would present as upper extremity flaccidity followed by spasticity. Sensory involvement along with symptoms such as pain, dysthesia, and parasthesia is most common. Patients with TM may often demonstrate urinary urgency, bladder or bowel incontinence, bowel constipation, difficulty or inability to void, and finally, sexual dysfunction. When considering myelitis, urinary retention is often the first sign.
  - *Multiple sclerosis:* The most common urinary symptom that patients with MS complain of is urinary urgency. Urodynamic studies of MS patients have shown underlying detrusor hyperreflexia is the cause of this urinary urgency [15].
- Structural
  - *Cervical myelopathy:* The most common urodynamic finding in cervical myelopathy is a variable combination of detrusor hyperreflexia and detrusor sphincter dyssynergia. Most of these patients will demonstrate long tract symptoms and signs. However, neurogenic features associated with voiding disorder, such as urinary hesitancy, poor stream, and incomplete emptying, can appear similar to features that occur due to an obstructed [18] outlet and are easily confused.
  - *Syringomyelia:* Syringomyelia may be an incidental finding initially but can later lead to spinal cord dysfunction from fluid expansion in the central spinal canal. Typically, spinothalamic tract neurons are compressed as they decussate in the anterior white commissure,; however, the pyramidal tract can also be impacted, causing detrusor hyperreflexia. The dorsal column is typically spared in syringomyelia [23].
- Infectious
  - *Tropical spastic paraparesis:* Tropical spastic paraparesi*s* is a disease of progressive myelopathy caused by HTLV1 virus infection [24]. The disease progresses slowly over the course of a decade before having onset usually before age 40. Back pain is often prominent [19]. An early and often presenting feature of this disease is urge incontinence due to detrusor hyperreflexia [20], which is also the most common urodynamic abnormality in tropical spastic paraparesis.
  - *Neurosyphilis:* Neurosyphilis is now rarely seen, though it was once a common cause of bladder dysfunction. Tabes dorsalis commonly leads to an areflexic and hyposensitive bladder that is the result of dorsal column and root involvement [25]. A range of abnormal urodynamic findings have been reported in patients with Tabes dorsalis [26].

### 4.6. Cauda Equina and Bladder Control

Second order parasympathetic innervation running from the cauda equina to the detrusor terminates in parasympathetic ganglia in the bladder well. Therefore, when there is damage to cauda equina, the detrusor is decentralized and not denervated, and sympathetic innervation of the bladder neck may still remain. Typically, patients with cauda equina syndrome present with a combination of symptoms including the loss of voluntary control of the anal and urethral sphincter and perineal sensory loss. Damage to the S2–S4 roots is typically the cause of the perineal sensory loss. Loss of sexual responsiveness is also often present. Various [21] bladder dysfunction conditions have been reported in patients who have cauda equina lesions, including detrusor hyperreflexia.

### 4.7. Peripheral Innervation and Bladder Control

- *Guillain-Barré syndrome:* GBS is an acute distal autonomic neuropathy. About one-quarter of patients with GBS have bladder symptoms. Both detrusor areflexia and bladder overactivity have been noted. Painful urinary retention usually occurs in both cholinergic and pan dysautonomia.
- *Pelvic nerve injuries:* Peripheral innervation of pelvic organs may be damaged by extirpative visceral surgery, such as resection of rectal carcinoma, radical prostatectomy, or radical hysterectomy. A prospective study of patients undergoing sphincter sparing surgery for low rectal carcinomas in which each patient acted as their own control showed that post-operatively there was a significant increase in post-micturition residual urine volume. In 15%, there appeared to have been severe damage to the parasympathetic innervation of the detrusor, resulting in long-term painless retention, a poor stream, loss of normal bladder sensation during filling, and near loss of detrusor contraction pressure [27] (Table 1).

## 5. West Nile Virus

### 5.1. West Nile Virus Biochemical Profile and Pathogenesis

West Nile virus (WNV) is part of the family of viruses called Flaviviridae, which includes dengue, yellow fever, and Japanese encephalitis [28]. The virus was one of the first arthropod-borne viruses (arboviruses) identified. It was first isolated from a patient presenting with fever in the West Nile district of Uganda in 1937.

WNV is an enveloped, spherical virus about 30–50 nm in diameter. It consists of a nucleocapsid [29] core surrounded by a lipid bilayer membrane and is a positive-stranded RNA virus with an 11-kilobase genome. There are five lineages of WNV and lineage I is predominant in North America, specifically clade 1a. Human WNV infections typically spread from culex mosquitoes.

Dendritic cells are infected and transport the virus in draining lymph. Infection of the CNS is typically associated with high levels of viremia, and results from either transport along peripheral neurons or disruption of the blood–brain barrier. It is thought that replication-dependent apoptosis causes neuron death and can lead to neuroinvasive disease [30].

### 5.2. Epidemiology

A group of meningoencephalitis cases associated with muscle weakness was observed in August 199 in New York City, NY. [31]. Initial investigations suggested that an arthropod-borne virus (arbovirus) may be the cause, and as such, serologic and viral testing were conducted to attempt to identify this potential cause [32].

In 2000, it was discovered that WNV survived in overwintering culex mosquitos in New York. This finding ruined initial hopes that WNV would not be able to thrive in more temperate climates and predicted the westward spread of WNV [33].

### 5.3. Related Neurological Disorders

Approximately 5% of patients develop neurologic symptoms which include meningitis, encephalitis, and poliomyelitis-like disease (flaccid paralysis). The fatality rate for patients with neurologic disease is approximately 10% [30].

The neuroinvasive disease, when it occurs, is frequently meningitis [34]. However, children with WNV have also been associated with poliomyelitis, fatal encephalitis, rhombencephalitis, and hepatitis. Additionally, just as seen in adult populations, immunocompromised children [35] are at greater risk of severe illness.

- *West Nile meningitis* is clinically similar to other viral meningitides, with the abrupt onset of fever and headache, as well as meningeal signs such as nuchal rigidity, Kernig’s and/or Brudzinski’s signs, and photophobia or phonophobia. The associated headache may be severe, requiring hospitalization for pain control. The associated gastrointestinal disturbance may result in dehydration, exacerbating head pain, and systemic symptoms [36].
- *West Nile encephalitis* may range in severity from a mild, self-limited confused state to severe encephalopathy, coma, and death. Several neurological syndromes, primarily extrapyramidal disorders, have been observed in patients with West Nile encephalitis [37]. Increased intracranial pressure and cerebral edema are infrequently associated with West Nile encephalitis.
- *West Nile encephalitis* generally develops soon after illness onset, usually within the first 24 to 48 h. Acute flaccid paralysis generally develops rapidly and may be abrupt, occasionally raising clinical concerns of stroke [38]. The weakness is usually asymmetric and often results in monoplegia. Patients with severe and extensive spinal cord involvement develop more symmetric dense quadriplegia. Central facial weakness can also be seen. Sensory loss or numbness is generally absent, though some patients experience intense pain in the affected limbs just before or during the onset of weakness, and this limb pain may be persistent [39]. In particular, quadriplegia and respiratory failure are associated with high morbidity and mortality, and recovery is slow and incomplete. More than 50% of the mortality associated with WNV-associated acute flaccid myelitis occurs in patients with acute neuromuscular respiratory failure.

## 6. Differential Diagnoses and Neuroimaging

### 6.1. Differential Diagnoses of Urinary Retention with Meningoencephalitis and Myeloencephalitis

Our case is a rare case of meningoencephalitis and myeloencephalitis presenting as an acute urinary retention. This combination of presentations is termed Elsberg syndrome, which is another differential diagnosis of meningitis retention syndrome (MRS). Sacral herpes, a variant of GBS, typical ADEM, typical MS, NMO, myelitis with leg weakness, herpetic brainstem encephalitis, chemical meningitis secondary to focal subarachnoid bleeding, and other causes of neurologic urinary retention should be excluded when making an MRS diagnosis. A detailed history, MRI of the brain and spinal cord, and nerve conduction studies are a few tests necessary to make the differential diagnosis.

Urodynamic studies in patients with MRS/Elsberg syndrome showed a contractile type of neurogenic bladder with retention and impaired sensation during the filling phase. It is caused by an underactive detrusor muscle that is unable to reach the intravesical pressures needed to begin micturition [40]. Neurogenic bladder can be related to neurological lesions anywhere along the neurogenic axis. Underactive detrusor due to a lower motor neuron lesion can be seen in neurological disorders such as Elsberg syndrome (infectious sacral polyradiculitis). Its presentation mimics MRS [8] but differs in some key aspects. Elsberg syndrome is usually caused by a herpetic viral infection that leads to myeloradiculitis without meningitis. Fever may also be absent. Due to the lack of neurological signs and symptoms of meningitis, CSF analysis results for Elsberg syndrome are not as significant compared with those of MRS. Symptoms of Elsberg syndrome not seen in MRS include muscle weakness, lower back pain due to radiculitis, herpetic genital vesicles, and hypoesthesia. Hyperintense T2 lesions on spinal cord MRI may represent infectious pathologies in Elsberg syndrome patients. One such example of an infectious pathology would be activation of the herpes virus in the dorsal root ganglia with axonal spreading to the spinal cord. In comparison, a spinal cord MRI demonstrates no lesions in MRS. However, there is some speculation that there may be reversible lesions in the corpus callosum splenium [41] (Table 2).

Occasionally, there are cases of underactive bladder caused by upper motor neuron lesions in the brain or spinal cord. ADEM is an example for this condition. The clinical manifestations of ADEM cases differ markedly from those of MRS, as it presents with encephalitic signs such as disturbance of consciousness, seizure, or aphasia, as well as myelitic signs such as gait abnormalities and sensory level abnormalities additional to aseptic meningitis signs. MRS lacks encephalitic signs [42,43]. Brain and spinal cords are common in ADEM, but not seen in MRS. Yet recently, a reversible splenial lesion was described in a MRS patient’s brain MRI [41]. It has been suggested that MRS may be a milder variant of ADEM.

Lack of leg paresthesias can help differentiate GBS, polyneuropathies, and conditions that affect the lower motor neurons from MRS.

### 6.2. Neuroimaging Findings and Pathways Affected Leading to Urinary Retention

An early review of central bladder control and functional imaging suggests that the most important regions of interest during full and empty bladder are the anterior cingulate gyrus, PAG, pons, insula, prefrontal cortex [44], and spinal cord fibers and associated interneurons. Urinary retention issues may be secondary to many different causes, one of which is voiding dysfunction, which is a urinary condition where the bladder muscle and urethra are uncoordinated. Neuroimaging of women with urethral sphincter dysfunction causing urinary retention have shown peripheral nerve neuromodulation associated with an altered right postcentral gyrus as well as precentral/temporal and inferior temporal region activity [45]. Urinary retention has also been found to be associated with lesions in dorsal tegmentum of the medulla and pontomedullary junction on both sides, though this does not seem to be common [46].

## 7. Summary and Conclusions

### 7.1. Case Summary

A 9-year-old girl presented to the emergency department with mixed Elsberg syndrome and meningitis retention syndrome symptoms after suffering from mosquito bites to her back two weeks prior. The symptoms included nausea, tachycardia, tachypnea, lethargy, somnolence, low-grade fever, scattered rash, bilateral weakness in the upper extremities, and severe urinary retention. This patient steadily experienced worsened symptoms until finally receiving PLEX treatment, after which she quickly improved.

Post-WNV infection can lead to encephalomyelitis presentation in pediatric population. It may present as meningitis retention syndrome with symptoms of urinary retention, subacute encephalopathy, ophthalmoplegia, and paraparesis secondary to the spinal cord, brainstem, and cortical inflammation. The patient may suffer symptoms of an increased intracranial pressure that may need aggressive invasive treatment with a shunt such as a lumbar drain. Intravenous immunoglobulins and methylprednisolone treatments were not successful in our patient. It is likely that the aggressive treatment with plasma exchange was the most effective in relieving the symptoms.

A few differential diagnoses for this case include Elsberg syndrome, meningitis retention syndrome, autoimmune encephalomyelitis, GBS, Bickerstaff encephalitis, and ADEM. Serology testing and imaging such as MRI were critical for investigating causes and identifying treatment modalities. Given that that the patient tested positive for West Nile virus, as well as her symptoms and her history of being bitten by mosquitoes, it is most likely that West Nile virus caused these severe neurological and urinary changes.

### 7.2. Conclusions and Future Directions

Elsberg syndrome is a syndrome that is typically caused by an infection that then causes acute or subacute lumbosacral radiculitis bilaterally. It may also cause lower spinal cord myelitis at times, and patients will typically present with a wide range of neurological symptoms. Currently, the literature on Elsberg syndrome, particularly that in pediatric populations, is limited. To the best of our knowledge, this is the first reported case of its kind. Since Elsberg syndrome may have many different differentials, cases as such will help reveal best practices for the identification, work-up, and treatment of Elsberg syndrome in pediatric populations. Further research can continue to report on similar cases and provide additional direction about how to best provide care for pediatric patients with rarer conditions that manifest in altered neurogenic control of urinary functioning.

## Figures and Tables

**Table 1:** Table of pathologies that may cause bladder control issues, their locations, and associated symptoms/features [19,21,22].

Pathologies	Localization	Associated Symptoms/Features
Transverse myelitis	Spinal cord	Bowel and bladder dysfunction, rapid onset weakness, and sensory deficits
Multiple sclerosis	Central nervous system/spinal cord	Urgency and detrusor hyperreflexia
Cervical myelopathy	Spinal cord	Detrusor hyperreflexia and detrusor sphincter dyssynergia
Syringomyelia	Spinal cord	Can cause progressive spinal cord dysfunction
Tropical spastic paraparesis	Spinal cord	Back pain and detrusor hyperreflexia
Neurosyphilis	Brain/spinal cord	Areflexic and hyposensitive bladder
Diabetic cystopathy	Peripheral nervous system	Often asymptomatic
Guillain–Barré syndrome	Peripheral nervous system	Detrusor areflexia, bladder overactivity, and can cause painful retention
Pelvic nerve injury	Peripheral nervous system	Poor stream, loss of normal bladder sensation, and lasting painless retention
Myotonic dystrophy	Skeletal and smooth muscle	Involves bladder smooth muscle

**Table 2:** Comparison table of Elsberg syndrome and meningitis retention syndrome presentations [40,42].

	Elsberg Syndrome	Meningitis Retention Syndrome
Urinary retention/constipation/pelvic floor dysfunction	+	+
Impaired sensation during filling phase	+	+
Underactive detrusor muscle	+	+
Meningitis symptoms	+/−	+
Fever	+/−	+
CSF analysis	+/−	+
Herpetic skin lesion	+	−
Sensory paresthesias/hypoesthesia	+	−
Muscle weakness	+	−
Lower back pain due to radiculitis	+	−
Evidence of lumbosacral radiculitis with concomitant thoracic and lumbosacral myelitis on neuroimaging	+	−

## Data Availability

No new data were created or analyzed in this study. Data sharing is not applicable to this article.
